# Continuous Glucose Monitoring Use in the Management of Type 2 Diabetes in Primary Care: Cross-Sectional Survey of Provider Comfort

**DOI:** 10.2196/92196

**Published:** 2026-06-04

**Authors:** Ryley Harris, Catherine Guariglia, Amy Cunningham

**Affiliations:** 1 Department of Family and Community Medicine, Thomas Jefferson University, 1015 Walnut Street, Suite 411, Philadelphia, PA, 19107, United States, 1 215 955 0535; 2 Department of Family and Community Medicine, Cooper University Hospital and Cooper Medical School of Rowan University, Camden, New Jersey, United States

**Keywords:** diabetes, type 2 diabetes, CGM, continuous glucose monitor, primary care, PCP, diabetes technology, patient education, primary care provider

## Abstract

This study assessed primary care providers’ comfort with prescribing and using continuous glucose monitoring technology for type 2 diabetes management, identifying key barriers and educational needs.

## Introduction

Diabetes affects approximately 40.1 million Americans, with the burden of care increasingly falling on primary care providers (PCPs), particularly in rural areas with limited endocrinologist access [1,2]. Continuous glucose monitoring (CGM) has demonstrated superior glycated hemoglobin (HbA_1c_) reductions and reduced hypoglycemia exposure compared to blood glucose monitoring [3,4]. The American Diabetes Association now recommends CGM for individuals with type 2 diabetes using daily insulin injections [5]. However, little is known about PCPs’ comfort and experience with this tool in routine visits. Two surveys have found varied levels of CGM experience, high PCP receptivity, and a need for education and practice support [6,7]. This study adds to the existing evidence by assessing PCPs’ comfort with CGM prescribing and needed resources; findings will inform future interventions.

## Methods

### Study Design

An original survey was developed and included demographic questions (race, gender, clinical role, years since training, practice setting), diabetes patient percentage, CGM experience, and 12 Likert-scale questions evaluating comfort with CGM benefits, patient education, prescribing, and interest in additional training (the full survey is available in Multimedia Appendix 1). The survey was distributed electronically via Qualtrics to PCPs (internal medicine physicians, family medicine physicians, nurse practitioners, and physician assistants) at 6 geographically diverse primary care sites within the Jefferson Primary Care Network, all associated with residency programs. The survey remained open for 10 weeks from May 2024 to July 2024 with one follow-up reminder. Data were analyzed using SPSS (version 30; IBM Corp) with descriptive statistics; inferential tests were not performed due to sample size limitations.

### Ethical Considerations

The project received exemption from a full institutional review board review by the Office of Human Research Institutional Review Board at Thomas Jefferson University on April 3, 2024, under Exemption Category 2: Research Involving Educational Tests, Surveys, Interviews, or Observation of Public Behavior, pursuant to Title 45 Code of Federal Regulations Part 46.104(d) [8].

## Results

The response rate was 38% (64/168 surveys). Half of the respondents (32/64) had prescribed CGM devices infrequently, 25% (16/64) regularly prescribed CGM, 22% (14/64) had managed patients using CGM without prescribing, and 2 of 64 (3%) had never managed a patient using CGM. Where percentages represent combined responses, multiple categories from the figures were summed to obtain the values reported (eg, “extremely comfortable” and “somewhat comfortable” were combined into a single comfort category).

**Figure 1.**
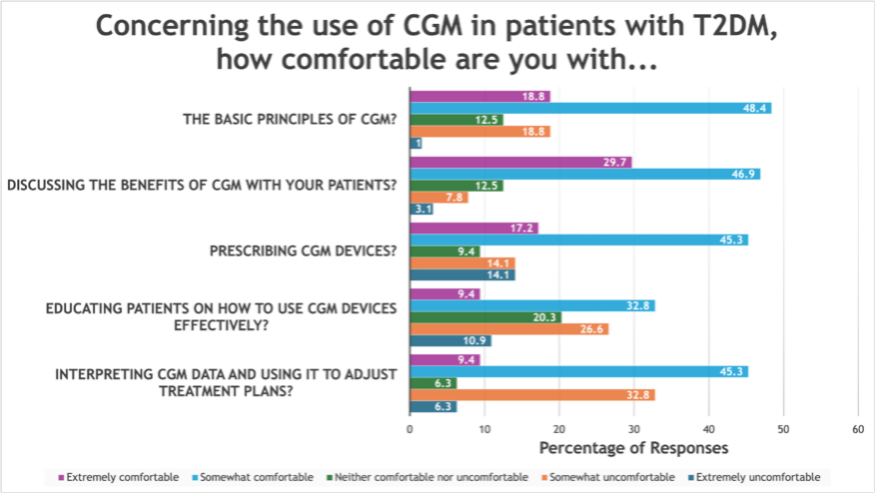
PCPs’ comfort levels with various aspects of CGM use. While 43 of 64 providers (67%) are comfortable with CGM basics and 49 of 64 (74%) can discuss its benefits with patients, fewer feel confident with more specialized aspects, such as prescribing (40/64, 63%), interpreting data (35/64, 55%), and educating patients (27/64, 42%). CGM: continuous glucose monitoring; PCP: primary care provider.

Forty-three of 64 respondents (67%) reported comfort understanding basic CGM principles, and 49 (77%) felt comfortable discussing CGM benefits with patients. Most PCPs felt comfortable prescribing CGM devices (40/64, 63%). However, fewer expressed comfort educating patients effectively on CGM use (27/64, 42%) or interpreting CGM data to adjust treatment plans (35/64, 55%) (Figure 1).

**Figure 2.**
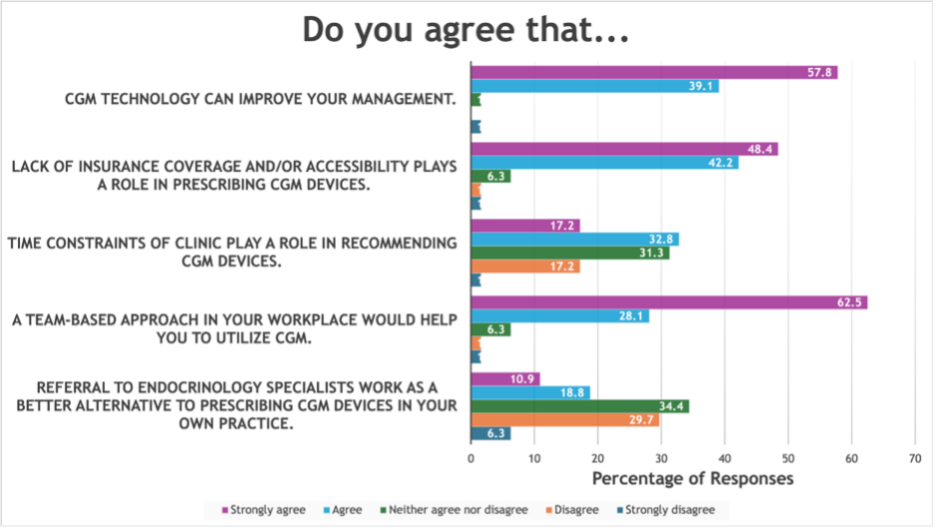
Barriers and facilitators to CGM adoption in primary care. Providers see CGM as valuable, but barriers like insurance (58/64, 91%) and time constraints (32/64, 50%) hinder its use. Fifty-eight of 64 providers (91%) favor a team-based approach to support CGM implementation, indicating collaboration could improve CGM accessibility and utilization. CGM: continuous glucose monitoring.

While 62 of 64 respondents (97%) agreed CGM improves PCP diabetes management, 58 of 64 (91%) identified insurance coverage as a significant prescribing barrier, and 32 of 64 (50%) reported time constraints as an additional barrier. Notably, 58 of 64 (91%) agreed a team-based approach would support CGM integration, while only 19 of 64 (30%) believed endocrinology referral would be preferable to prescribing CGM in their own practice (Figure 2).

Interest in further training was high, with 57 of 64 respondents (89%) expressing significant interest in receiving additional CGM education, and 62 of 64 (97%) indicating strong interest in using CGM more frequently in their care of patients with type 2 diabetes.

## Discussion

This study expands the evidence base on PCP comfort and experience with CGMs. While most PCPs felt comfortable discussing CGM benefits and prescribing devices, less felt confident with patient education or data interpretation. These findings align with previous studies suggesting a lack of training on emerging technologies prevents optimal primary care use [2,5-7].

Identified barriers, such as insurance coverage and limited visit time, emphasize systemic challenges hindering CGM adoption, consistent with a recent survey of Veterans Affairs PCPs [7]. Surprisingly, nearly one-third of respondents were neutral on the role of time constraints—this could reflect lack of experience prescribing CGMs or suggest that respondents have found ways to address time barriers. This item requires further exploration in follow-up studies.

The high interest in additional training presents an opportunity for targeted interventions, such as provider education modules. Team-based approaches, supported by 91% (58/64) of respondents, could alleviate PCP burden by incorporating pharmacists or other health care professionals in CGM education and management. Our survey is the first to assess PCP attitudes toward endocrinology referrals for CGM. Interestingly, most respondents did not agree with relying on endocrinology referrals for CGM adoption, with 22 of 64 (35%) disagreeing/strongly disagreeing, and 22 of 64 (35%) neutral. The high percentages of disagreement/neutrality are likely due to limited endocrinologist availability and therefore limited experience working with endocrinologists. These findings emphasize the importance of PCP CGM education and interdisciplinary team development. Recent pilots have tested provider and staff education to improve PCPs’ CGM uptake; results have included increased CGM knowledge [9], improved PCP comfort with CGM, and increased CGM prescribing [10]. One quality improvement study used an interdisciplinary CGM team to increase patient use, resulting in increased patient time in HbA_1c_ range [11]. These findings, along with the results of our survey, will be used to develop local pilot interventions.

Limitations include sampling within one health care network, potential selection bias due to a low response rate, and lack of statistical power to explore differences by provider characteristics. We also did not collect practice data. Future research should examine larger, multisystem samples, examine practice context, and evaluate the impact of CGM intervention on provider knowledge, attitudes, prescribing behavior, and patient outcomes.

By addressing challenges through targeted training and team-based approaches, PCPs will be empowered with the skills and resources needed to use CGM to improve patient outcomes.

## Supplementary material

10.2196/92196 Multimedia Appendix 1 Survey questions assessing primary care provider comfort and experience with continuous glucose monitoring.

## Abbreviations

CGM: continuous glucose monitoring
HbA_1c_: glycated hemoglobin
PCP: primary care provider
