# Supplementary material for: Continuous Glucose Monitoring Use in the Management of Type 2 Diabetes in Primary Care: Cross-Sectional Survey of Provider Comfort
Source: JMIR Form Res. 2026 Jun 4;10:e92196. doi: 10.2196/92196 (PMC13235701; doi:10.2196/92196)
Supplement: Multimedia Appendix 1 [file formative-v10-e92196-s001.pdf]

Provider Comfort CGM

Start of Block: Default Question Block

Q1 Which of the following best defines your race or ethnicity? Select all that apply:

- ☐ Hispanic/Latino/a/Spanish Origin (1)
- ☐ American Indian/Alaska Native/Indigenous (2)
- ☐ Asian (3)
- ☐ Black/African American (4)
- ☐ Native Hawaiian/Pacific Islander (5)
- ☐ White (6)
- ☐ Middle Eastern/North African (7)
- ☐ Other (8)

Q2 What is your current gender identity? Select all that apply:

- ☐ Female/Woman (1)
- ☐ Male/Man (2)
- ☐ Genderqueer/Gender non-conforming (3)
- ☐ Non-binary (4)
- ☐ Choose not to disclose (5)
- ☐ Prefer to self-describe (please specify) (6) ____

Q3 Which best describes your professional role:

- ○ Attending, faculty, or community physician (1)
- ○ Resident (2)
- ○ Physician Assistant (3)
- ○ Nurse Practitioner (4)
- ○ Other (please specify) (5) ____

Q4 Which best describes your medical specialty:

- ○ Family Medicine (1)
- ○ Internal Medicine (2)
- ○ General Pediatrics (3)
- ○ Internal Medicine-Pediatrics (4)
- ○ Other (please specify) (5) ____

Q5 How much of your week is spent clinically?

- ○ 0-20% (1)
- ○ 21-40% (2)
- ○ 41-60% (3)
- ○ 61-80% (4)
- ○ 81-100% (5)

Page Break

Q6 How many years has it been since you completed training?

- ○ Still in training (1)
- ○ 1-5 years (2)
- ○ 6-10 year (3)
- ○ 11-15 years (4)
- ○ 16-20 years (5)
- ○ More than 20 years (6)

Q7 In what setting do you typically practice?

- ○ Urban, academic (1)
- ○ Urban, community (2)
- ○ Suburban, academic (3)
- ○ Suburban, community (4)
- ○ Rural, academic (5)
- ○ Rural, community (6)

Q8 Estimate the percentage of patients with Type 2 Diabetes Mellitus (T2DM) in your practice?

- ○ 0-20% (1)
- ○ 21-40% (2)
- ○ 41-60% (3)
- ○ 61-80% (4)
- ○ 81-100% (5)

Q9 Which statement best describes your experience with Continuous Glucose Monitors (CGM)?

- ○ I have never heard of CGM (1)
- ○ I have heard of CGM, but I have never had patients on one (2)
- ○ I have had patients on a CGM, but have never prescribed one (3)
- ○ I have prescribed a CGM, but not often (4)
- ○ I regularly prescribe CGM in my practice (5)

Page Break

Q10 Concerning the use of CGM in patients with T2DM, how comfortable are you with...

	Extremely uncomfortable (1)	Somewhat uncomfortable (2)	Neither comfortable nor uncomfortable (3)	Somewhat comfortable (4)	Extremely comfortable (5)
the basic principles of CGM? (1)	○	○	○	○	○
discussing the benefits of CGM with your patients? (2)	○	○	○	○	○
prescribing CGM devices? (3)	○	○	○	○	○
educating patients on how to use CGM devices effectively? (4)	○	○	○	○	○
interpreting CGM data and using it to adjust treatment plans? (5)	○	○	○	○	○

Page Break

Q11 Concerning the use of CGM in patients with T2DM, how much do agree or disagree with the following statements?

	Strongly disagree (1)	Disagree (2)	Neither agree nor disagree (3)	Agree (4)	Strongly agree (5)
CGM technology can improve your management. (1)	○	○	○	○	○
Lack of insurance coverage and/or accessibility plays a role in prescribing CGM devices. (2)	○	○	○	○	○
Time constraints of clinic play a role in recommending CGM devices. (3)	○	○	○	○	○
A team-based approach in your workplace would help you to utilize CGM. (4)	○	○	○	○	○
Referral to endocrinology specialists work as a better alternative to prescribing CGM devices in your own practice. (5)	○	○	○	○	○

Page Break

Q12 Concerning the use of CGM in patients with T2DM, how interested are you in...

	Not at all interested (1)	Slightly interested (2)	Somewhat interested (3)	Moderately interested (4)	Extremely interested (5)
utilizing CGM in your care for patients with T2DM? (1)	○	○	○	○	○
receiving additional training or resources to enhance your knowledge about utilizing this technology? (2)	○	○	○	○	○

End of Block: Default Question Block
